# Machine Learning and Health Science Research: Tutorial

**DOI:** 10.2196/50890

**Published:** 2024-01-30

**Authors:** Hunyong Cho, Jane She, Daniel De Marchi, Helal El-Zaatari, Edward L Barnes, Anna R Kahkoska, Michael R Kosorok, Arti V Virkud

**Affiliations:** 1 Department of Biostatistics University of North Carolina at Chapel Hill Chapel Hill, NC United States; 2 Division of Gastroenterology and Hepatology University of North Carolina at Chapel Hill Chapel Hill, NC United States; 3 Center for Gastrointestinal Biology and Diseases University of North Carolina at Chapel Hill Chapel Hill, NC United States; 4 Department of Nutrition University of North Carolina at Chapel Hill Chapel Hill, NC United States; 5 Division of Endocrinology and Metabolism University of North Carolina at Chapel Hill Chapel Hill, NC United States; 6 Center for Aging and Health University of North Carolina at Chapel Hill Chapel Hill, NC United States; 7 Kidney Center University of North Carolina at Chapel Hill Chapel Hill, NC United States

**Keywords:** health science researcher, machine learning pipeline, machine learning, medical machine learning, precision medicine, reproducibility, unsupervised learning

## Abstract

Machine learning (ML) has seen impressive growth in health science research due to its capacity for handling complex data to perform a range of tasks, including unsupervised learning, supervised learning, and reinforcement learning. To aid health science researchers in understanding the strengths and limitations of ML and to facilitate its integration into their studies, we present here a guideline for integrating ML into an analysis through a structured framework, covering steps from framing a research question to study design and analysis techniques for specialized data types.

## Introduction

As a brief overview, machine learning (ML) is generally characterized by model complexity and capacity for processing high-dimensional or complicated data forms and is often mentioned as an antonym to traditional statistical learning algorithms. However, this division is not clear, and ML algorithms range from traditional statistical analysis tools such as simple linear regression to cutting-edge deep neural network algorithms. While often used interchangeably with artificial intelligence (AI), ML is a subset of AI and seeks to use data-driven methods to identify patterns and make decisions. This can then be used in the field of AI to allow problem-solving and decision-making.

ML is becoming increasingly popular in the research community due to the proliferation of complex or unstructured data sets and the increased capacity and access to computing power needed to run these models. ML models can often discover sophisticated and surprising patterns in these data sets that would be difficult to discover using classical methods [1,2]. The health science research domain has been no exception to this paradigm, as the health science fields have an abundance of data well suited for these models, such as genomics sequencing data and electronic health records (EHR) data [3-6]. Applications of ML to the health field can lead to targeted interventions to provide support for health care professionals [7]. ML has also become almost indispensable to the fast-growing field of PM, which uses rich patient information to precisely target interventions [8].

**Figure 1:**
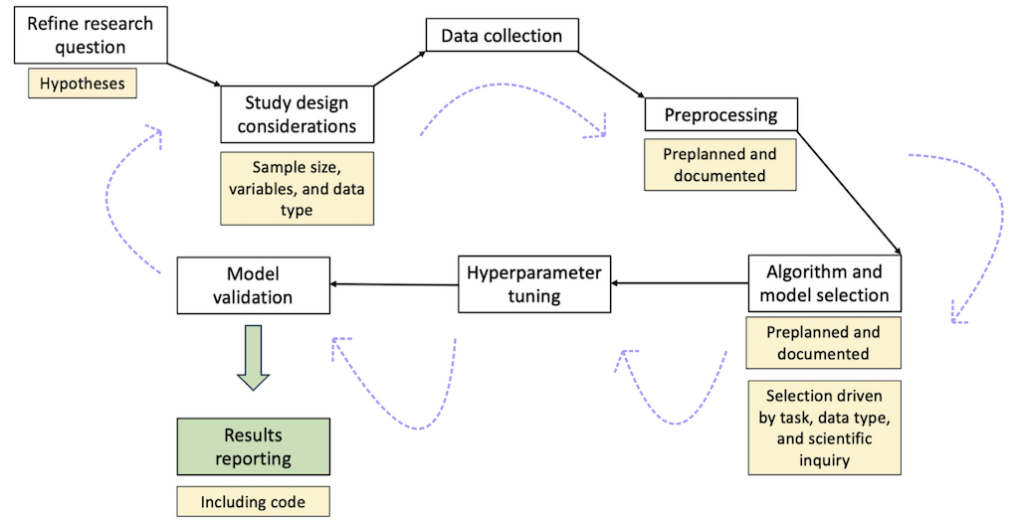
Machine learning workflow for a health science research question, from research question refinement to results reporting, with additional considerations. The cyclic nature of the process is reflected in the arrows, as several different iterations may be considered before narrowing down to a decisive pipeline, leading to result reporting.

This paper provides a sequential framework for health scientists intending to use ML in a research proposal and discusses types of analyses that can be done and factors to consider. It will also include a special introduction to the field of PM, which has become a popular research area with the development of new ML methodologies. Finally, we discuss some unique data types and analysis techniques specific to those application areas. In general, throughout the study design process, documentation and preplanning are highly recommended for the sake of reproducibility of the work carried out. For a visual illustration of the research pipeline flowchart, see Figure 1. There are also existing pipelines such as MLOps and CRISP used in business and industry settings that may be adapted to health science research fields; however, this paper will follow a framework more commonly seen in health science research. We relegate some technical topics, such as general sample size calculations, model training, and model tuning and validation to Multimedia Appendix 1 [9-21]. Readers are also encouraged to reference other ML primers, such as one for epidemiologists [22] and 1 for biologists [23].

## Experimentation

This section introduces step-by-step core considerations for designing an ML-involved research project.

### Refining Research Questions: What Can Machine Learning Do?

ML methods can be used to answer questions for studies that may fall within the following categories: prediction, estimation, understanding causal associations, and decision support. ML can also help support main analyses as an auxiliary tool through missing data imputation, inverse propensity score weighting, dimensionality reduction, and variable selection. This last point will be covered further in the Data Collection and Preprocessing section.

Inquiries in traditional studies are often limited to the discovery and measurement of the size of certain effects or to establishing the causal relationship between variables. These are called estimation and causal inference, respectively. The recent realm of research has also expanded to prediction [24], where algorithms can predict an outcome for a patient when given a set of input variables. Functionalities such as estimation, establishing causal relationships, and prediction are meant to, in an indirect manner, support clinical decision-making. However, there are decision-support frameworks that explicitly provide recommendations through reinforcement learning (RL; defined in the section Advanced Concepts). One of the most important applications in the medical domain is PM, where the goal is to provide an optimal treatment for individual patients each with unique characteristics [8].

### Prediction

As a concrete example, suppose a researcher is interested in investigating the health effects of electronic screen time use over several months [25]. This is an example of a study where the research question hinges on accurate prediction of the use of a screen. Since it is impractical and unethical to monitor an individual for several months and self-reported measures can be unreliable, the use of ML algorithms for predicting screen time is useful [26]. Prediction has also been used in the identification of early cancer diagnoses using image data analysis [27]. Classification of patients for disease screening, a prediction task, can be performed with high accuracy using ML.

Although the accuracy of a predictive algorithm is considered one of the most important virtues, interpretability [28] is another important aspect to consider, especially in health science research. Interpretability often comes at the price of reduced accuracy, which is sometimes framed as the “interpretability-accuracy trade-off.” More complex models, which may improve prediction accuracy, maybe less interpretable, as it can be difficult to trace why the model arrived at such a decision, how the predictors relate to the outcome, and how to interpret the results. Interpretability generally is used to mean being able to understand the inner workings of a model, but as evidenced by the previous sentence, it can encompass several different aspects. These can range from overall model structure, ability to explain individual predictors, transparency of decision-making processes, and more. Measuring interpretability is a challenge, as it can be context-dependent for the problem you are working on; more information on interpretability can be found at [29]. In the screen time prediction example above, interpretability is not of concern but rather maximizing the prediction accuracy, as it may not be of interest how the algorithm predicted the values, but rather the predicted values themselves.

### Estimation

ML algorithms can also be used to estimate associations between exposures and health outcomes [30]. Examples include calculating the odds ratio of obesity while comparing 2 socioeconomic statuses, measuring the association between physical activity and mortality [31], and estimating the association between sociodemographic traits and diabetes prevalence [32]. However, the estimation procedures of ML algorithms are often limited to point estimation and usually lack inferential abilities such as *P* values and Bayes factors. Meaning, estimation procedures can usually find an approximate value of a parameter (like an average) through point estimation but are not usually able to output other quantities such as CIs or hypothesis tests which provide information on the population as a whole. This is because models that are nonparametric or complex may not make certain distributional assumptions, which makes quantifying CIs for a point estimate not easily doable. For an investigator wanting to confirm the positive effects of a medical treatment on patient health outcomes, ML often cannot discern whether the estimated effect size is statistically significant or not. Rather, this can be done through classical statistical tests, which possess inferential capabilities. However, this limitation is not the same as generating CIs for model performance, which is a separate procedure and generally more straightforward as model evaluation may involve data splitting or repeated sampling.

That being said, certain ML algorithms still have the potential for inferential capacity. Recently, a random forest-based framework for judging the statistical significance of heterogeneous treatment effects for individuals with specific covariate values has been developed [33]. Additionally, many other algorithms, such as support vector machine (SVM) and *k*-nearest neighbors (*k*-NN), can output CIs and *P* values for estimated effects [34,35]. However, these approaches are, in general, much less efficient than classical statistical tests and thus should be used after carefully considering the trade-off between flexibility (model specification) and efficiency (power of the test).

### Causal Inference

Understanding causal associations is the activity of investigating the cause of an outcome, such as the occurrence of disease. In the statistical literature, it is known as causal inference, which provides a foundation for establishing causality [36,37]. Research questions related to understanding causal associations include estimation of average or individual treatment effects (ATE and ITE, respectively) and identification of important risk factors or subgroups for a health outcome. A rich literature for causal inference methods has been developed in statistics. For example, when estimating average treatment effects [38] from observational data, propensity score matching [39] is frequently used, which is often done using flexible models such as random forests [40]. However, its use should be carefully considered due to potential small sample size issues and covariate imbalance.

### Study Design Considerations

Quality of data is a key design consideration for the successful use of ML. Given the complexity of ML, which often involves managing a vast range of input variables coming in various formats, it is crucial to plan the identification, collection, and management of these variables. Data from multiple sources—for example, clinical information, genomics data, and medical images have different dimensions—eventually needs to be aligned for downstream analyses using techniques such as feature concatenation, feature extraction, and tree and metric-based learning, so planning the process ahead of time is essential to consider any feasibility issues [41,42].

In ML studies, missing data are one of the most frequently observed issues that can harm the quality of data and can lead to bias. Thus, planning the data collection process to minimize missing data and setting up quality control checks on data entry errors is essential. Approaches to data missingness will be described in the Data Collection and Preprocessing section.

### Sample Size and Strategies for Sample Size Determination

In general, ML models with tunable parameters require much larger sample sizes than traditional statistical models to achieve the same level of estimation or prediction accuracy. Since ML models usually have much weaker model assumptions than traditional parametric models, when the dimension of a parameter is much larger, more data are needed for the estimator to determine the model structure on top of estimating the mean outcome or the parameters of interest. The phenomenon when the required sample size grows exponentially with the dimension of the parameter is called the “curse of dimensionality,” which is attributable to the nonparametric nature of ML models.

This relatively large sample size requirement is not the only issue, but precisely calibrating the required size is another challenge. Unlike traditional clinical trials, where the sample size of a study is planned to achieve a certain amount of power to detect a certain effect size [43,44], the sample size determination for ML has a different meaning, and there is no generic framework for it [45]. In ML, where the model performance is often measured in terms of prediction accuracy; measures, such as mean squared error and classification error rate, are meant to be controlled under a predefined level, and the sample size that meets such prediction accuracy is to be derived.

Popular choices of evaluation metrics include mean squared error and *R*^2^ for continuous outcomes, Brier scores for survival outcomes, classification error rate (accuracy rate) for categorical outcomes, and area under the receiver operating characteristics curve (AUC) for binary outcomes. However, these evaluation metrics should be chosen after consideration of the cost of wrong predictions and the benefits of correct predictions. For example, a model for predicting cancer may have to impose a higher cost for false negative than for false positive. Thus, a true negative rate (TNR) or a partial AUC could be considered for its evaluation measure after considering threshold selection and other possible reporting metrics. There are no “best” evaluation metrics, as this is highly dependent on the problem itself beyond the characterization of a classification or regression framework; differences in metrics can emerge when there are outliers in the data set, model comparison, and differential penalties for errors.

Although there is no deterministic sample size formula for predictive models, one can fit a learning curve on the training data for a given ML algorithm based on some evaluation measures such as the prediction error rate and AUC, which quantifies the overall accuracy of a binary classification model [45]. Essentially, the researcher is required to run the ML algorithm for the pilot data using training data and project the evaluation measure based on the fitted learning curve through the evaluation of the testing data. This evaluation measure is then used to inform the sample size or amount of data needed for the specific accuracy or statistical power desired [46].

To accurately estimate this curve, at least 2 or 3 points are required [47]. This means that the researcher is required to take at least 2 subsets of the available data and calculate 2 respective error rates. However, the pilot data might not capture all the biases present in the larger data set, as the sample may not be fully representative of the population or phenomena of interest. The researcher must be wary of generalizations using this pilot data. It is therefore recommended that a statistician trained in ML be present to assist with these technical sample size estimation procedures.

More details are included in section A of Multimedia Appendix 1 [9-21], which also includes information on how to mitigate the large sample requirement in neural networks through augmentation techniques and transfer learning (defined in the section Advanced Concepts).

### Data Collection and Data Preprocessing

As previously mentioned, EHR, administrative claims, clinical trials, and longitudinal cohort data are major data types in the ML world. However, there are also “specialized data types,” which require their own distinct methods of analysis due to their unique qualities. These include textual or language data, imaging, and genomics, and will be discussed in the Applications section. Due to the highly complex nature of the data being used, ML analyses often involve heavy data preprocessing. This step often requires more time than the main analysis itself and not only includes screening for erratic values, detecting and understanding outliers, and handling of missing values, but also transformation of the data into a software-friendly format, feature scaling, feature selection, dimensionality reduction, and sample splitting for validation, among others [48].

These procedures, while seemingly not important, may bring significant changes to the conclusion. For example, data preprocessing is an essential step for categorical features when using certain gradient boosting algorithms such as XGBoost, as the algorithm requires the categorical variables to be coded through mean coding or one hot coding before use in the model. Additionally, feature scaling would change the results of any methods involving Euclidean distance metrics such as principal component analysis (PCA), *k*-means, and *k*-NN.

As mentioned in the section Refining Research Questions: What Can Machine Learning Do? ML can be used as an auxiliary tool for missing data imputation [49], dimensionality reduction [50] before regression analysis, and variable selection, all of which can make an analysis more manageable.

Missing data, which typically arises in survival analysis, longitudinal studies, among other scientific studies, has great potential to create statistical bias if not accounted for in an auxiliary analysis [51]. Simply discarding observations with missing data may lead to selection bias and reduced sample size, resulting in incorrect estimation of relationships. Instead, the mechanism behind the missing data can be accounted for through an auxiliary analysis to mitigate the effects of the bias using tools such as imputation and maximum likelihood estimation. See, for example, “missForest” for imputation based on random forests [52]. As an example, [49] provides a real-use case of how ML methods can be used to impute missing data in a breast cancer problem.

### Algorithm and Model Selection

The choice of an ML method largely depends on the type of task and data type. For example, linear discriminant analysis (LDA) and *k*-means clustering can only be used with continuous predictors; SVM and support vector regression can be used for classification and regression problems, respectively; and random forests and neural networks are capable of both classification and regression. Table 1 lists commonly available algorithms in each category and summarizes their benefits and drawbacks.

**Figure 2:**
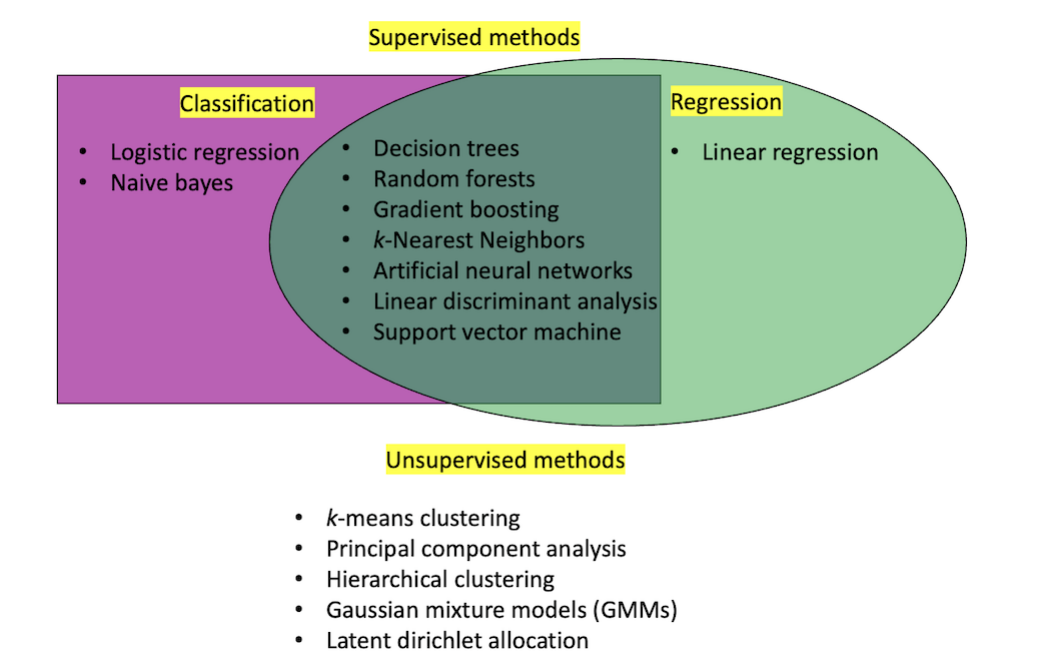
Commonly used algorithms in the supervised setting by algorithm type distinguished between classification and regression problems, as well as methods used in unsupervised learning.

Once the candidate algorithms are identified, the choice of the algorithm may be driven by the scientific inquiry, as discussed in the section Refining Research Questions: What Can Machine Learning Do? Additional factors for algorithm choice may include computing resources, data limitations, and data assumptions. Figure 2 gives a list of common ML algorithms and the purposes they may be used for. The nature of the scientific study will determine the importance of interpretability in the prediction of particular phenomena. A “black box” predictive model may not clearly explain why such predictions were made, only what the predictions are [53]. For clinicians who want to attribute a specific cause of an output, these methods may be less suited for their research question, and it is suggested to use a more interpretable model. As an extreme example, consider using an ML algorithm to support the decision of no amputation, minor amputation, or major amputation for a patient with diabetic foot ulcer. One can imagine that an interpretable ML algorithm must be preferred, as was proposed in [54], as the decision-making process needs to be clear before an amputation is carried out.

**Table 1:** Benefits and drawbacks of common machine learning methods in supervised and unsupervised settings. This list is not exhaustive and includes popular machine-learning algorithms in each category.

Methods			Strengths		Limitations
**Benefits and drawbacks of some supervised methods**					
	Logistic regression and linear regression	Interpretable Easy implementation		Overfitting with highly correlated data (use variable selection or shrinkage methods) Poor performance for nonlinearly separable data	
	Naive Bayes	Performs well even without conditional independence Easy implementation		Simple; outperformed by well-tuned, more complex models	
	*k*-nearest neighbors	Nonparametric (no model assumptions needed) High level of flexibility; performs well for nonlinear boundaries		Low interpretability Poor performance for high-dimensional data Difficulty dealing with missing values	
	Support vector machine	Performs well with high-dimensional data and nonlinear boundaries		Low interpretability Poor performance for imbalanced data Usually outperformed by newer methods	
	Decision trees	Interpretable for trivial data sets Nonparametric (no model assumptions needed) Works with nonlinear relationships Classification for more than 2 classes		Prone to overfitting Difficult to interpret for nontrivial data sets	
	Random forest	Handles high-dimensional data well Reduces overfitting from decision trees Reduces variance		Low interpretability Poor performance for sparse data	
	Gradient boosted trees	Increased accuracy over random forests		More difficult to implement due to tuning parameter selection	
	Artificial neural networks	Works well with many data types (images, text, audio, etc) Adaptable architecture		Low interpretability Overfitting if trained too long Requires a great deal of data	
**Benefits and drawbacks of some unsupervised methods**					
	*k*-means clustering	Fast, easy implementation		Only quantitative data No clear best way to choose k Poor performance for noncircular cluster	
	Hierarchical cluster	Reproducible Visually interpretable by dendrograms Cluster shape not assumed to be globular		Poor performance on high-dimensional data Hierarchy level must be selected	
	Gaussian mixture models	Flexibility since clusters can have irregular shapes No assumption of cluster number or level Accommodates mixed cluster membership		Poor performance on high-dimensional data	
	Linear discriminant analysis	Interpretable Can lower model variance over logistic regression if model assumptions are met		Can only be used with continuous predictors Poor performance for nonlinearly separable data (try quadratic discriminant analysis)	

Being too open-ended about model possibilities may lead to nonreproducibility or phenomena analogous to *P*-hacking, where researchers may choose the model that leads to the highest accuracy for the data at hand after trials of multiple approaches. This highlights the importance of having a held-out test set, which is used only at the end of model development to report model performance results, as well as having an appropriate justification for model selection. More details about a held-out test set can be found in the Hyperparameter and Model Validation section. It is important to be specific enough about the goals of the analysis to justify the use of different algorithms. At the same time, being too specific can put too many undesired constraints on research, unduly limiting the use of adequate algorithms and models [55].

### Hyperparameter Tuning and Model Validation

Assuming an appropriate model has been chosen, hyperparameter tuning and model validation become the next steps for an ML practitioner. In section B of Multimedia Appendix 1 [9-21], we provide guidelines for tuning hyperparameters for 2 popular ML methods—tree-based methods and neural networks. The relatively high performance of these models is achieved by adequately tuning the hyperparameters.

Model performance assessment metrics are used to determine how well a trained model performs on new, unseen data. Popular model assessment metrics include in regression: *R*^2^ values, mean absolute error, mean square error, and in classification: recall, *F*_1_-score, and AUC. Beyond these metrics, aspects of model performance can also include ease of use and deployment feasibility. In many health care cases, understanding how a model reached the conclusion as well as interpreting the results of the conclusion may be preferred over blackbox models, as medical decisions are made based on the results of the model. The model deployment aspect focuses on its practical use; a complicated model may not be used in resource-constrained settings, so its use may not be feasible.

Typically, the preprocessed data are split into separate training and testing sets. The term “validation set” is often used interchangeably with the term “test set” and usually refers to a portion of the data that are not used in training the model. The model is evaluated on the test set to give an unbiased estimate of model performance on unseen data. This test set cannot be touched before model fitting and is not used for training the model or tuning model parameters.

In some literature, the training data themselves may also be split into 2 separate data sets: one, dubbed the “training set,” is used to train the models to get parameter estimates, and the other, the “validation set,” is used to help tune parameters. Therefore, rather than a split into a training and testing set as previously mentioned, we have a training, validation, and testing split, where the test set is held out until model performance evaluation. The interchangeable use of “test set” and “validation set” in this case may be confusing, as they do not refer to the same thing when data are portioned this way—one must be careful in reading to understand which scenario is occurring.

When partitioning data, it is important for the test set to be representative of the data rather than having different characteristics than the training set. There are various factors to consider when forming a test set, which can depend on the use case. For example, one may want the training and test sets to contain records from different individuals for diagnosis purposes, or for the training and test sets to contain observations from different time points on the same individuals for prognosis. Saeb et al [56] discuss examples of using different types of splitting in cross-validation and how results can differ based on the partitioning.

Section B of Multimedia Appendix 1 [9-21] continues to discuss how to validate these models using *k*-fold cross-validation, which is a validation method that uses a given sample, assuming collecting additional data is difficult. For high-dimensional data where *k*-fold cross-validation is infeasible to implement due to computational costs, an alternative approach is introduced. It should be noted that cross-validation methods still require a held-out test set to evaluate model performance at the very end; *k*-fold cross-validation is used on the training data set to tune the parameters, but it does not replace the need for a separate testing set. An introduction to how ML models are trained is also discussed in section C of Multimedia Appendix 1 [9-21] for additional information and completion in the understanding of model training.

## Results and Reproducibility

The final step of any project is to report the results. Luo and colleagues [57] set up reporting standards of ML predictive model-based research for biomedical researchers, which include a list of reporting items to be included. Reporting of such items is essential to promoting reproducibility in research. Among the items are details including the nature of the study along with a background, objectives, clinical rationale, data sources, type of modeling, inclusion and exclusion criteria, time span, model validation strategies, handling of missing values, cleaning and transformation, candidate modeling techniques with justification, model selection criteria, clinical implications, and model limitations [57].

Several of these reporting items have been discussed in the paper and fall under the outlined categories of research question refinement, study design considerations, data collection and preprocessing, algorithm and model selection, hyperparameter tuning, and model validation. The goal of the list of reporting standards for predictive modeling is to encourage transparency and reproducibility to ensure credibility in the scientific community and the methodological soundness of research.

The need for transparent reporting is even more apparent when considering the nuances of ML. While studies involving ML have experimental design steps that overlap with general study considerations such as refining a research question, study design, and data collection, the use of ML in health science studies requires ML-specific considerations in terms of quality and size of data, adequacy of methods, and reproducibility. These issues are inherent in ML-involved research owing to both the complexity of data and ML models and a wide spectrum of ML methods. Readers are encouraged to read the literature [58-60] for guidelines on general study considerations. Therefore, there is a necessity for conscientious approaches to reporting.

Within each ML method, there are usually one or more hyperparameters, such as the depth and node size in tree methods and the penalty term in kernel regression [61]. Cherry-picking a hyperparameter after looking at the data multiple times may result in irreproducibility. This is why the held-out test set can only be used for result reporting and should not be used for further model development. As previously mentioned, for the sake of reproducibility, keeping documentation of the research pipeline from start to finish as outlined is also necessary.

## Application

This section includes specialized data types in ML.

### Natural Language Processing

Medical notes of physicians may contain important information beyond quantitative clinical records. They, however, are not readily analyzable without processing such as transcription and topic extraction. Natural language processing (NLP) does what was previously considered impossible by processing such nonstandard form of massive data into a readily analyzable format, opening huge opportunities for health science research.

NLP as a field has undergone a revolution since 2018. The seminal paper, “BERT” [62], delivered unprecedented performance on almost every major language task. NLP models using transformers (defined in the section Advanced Concepts), such as BERT and GPT [63,64], can be used for various language tasks, including classification, summarization, imputation, and prediction. The most common and useful tasks in medical NLP usually deal with hospital documents and patient interactions. For instance, NLP models can be used to automatically transcribe patient conversations, predict disease from medical notes, or impute missing values in medical forms [65]. There are many high-quality models trained on massive text corpora that can, out of the box, deliver state-of-the-art performance on almost any task.

Therefore, the first step in any NLP project is to select a pretrained model closest to the language domain being used and perform transfer learning. Transfer learning is where a model pretrained on 1 task is then trained on a related but different task. For example, for medical tasks, “Med-BERT: pretrained contextualized embeddings on large-scale structured EHR for disease prediction” and “SciBERT: a pretrained language model for scientific text” may be of use, as they are trained on similar language as is used in medical contexts [60,61,66]. The original BERT model will also work well for most purposes. The common structure of language and the size of most of these training data sets (terabytes for some models) mean that a general model will have almost certainly been exposed to any sort of text problem a researcher may be interested in due to the sheer breadth of data.

The next step is to preprocess the data so that text is converted into simple numeric tokens that can be used as inputs and process those tokens into small sets for the model to interpret. Once this is done, the model can be fine-tuned on the new data. This is done by taking the previously selected pretrained model and carefully training it with a low learning rate on the new data. Once this is done, the model should be ready for use. Extreme care needs to be taken to derive an optimal train and test split.

### Imaging

Imaging research has long been the most high-profile ML task. High-quality benchmark data sets such as the ImageNet challenge have provided robust methods for model assessment with useful pretrained networks for transfer learning [67,68]. The uses of imaging in biomedical applications are myriad. Diagnostics, such as automatic reading and classification of radiological scans or tissue biopsies, are an active area of research. Computer-assisted decision support, where ML algorithms mark anomalous areas for clinicians to investigate, is also relatively well developed. Each use case often requires highly specific knowledge and training data; we leave the specifics to clinical experts.

Convolutional neural networks are a type of deep learning model heavily used in image analyses, such as medical imaging. Convolutional neural networks can extract patterns from image pixels and are thus widely used in abnormality detection, segmentation, and classification [69].

For imaging, as with language, it is strongly encouraged to take advantage of transfer learning. High-quality models are available on many tasks. In addition, vision has demonstrated similar properties as language, and seemingly unconnected tasks often turn out to be very similar, such as categorizing pastries and segmenting tumors. Even if the images in question are very different sizes, it can still be effective to simply resize them to fit the network in question. As in all things, the best strategy is simply to experiment [70].

### Genomics

With its high-dimensional nature and the growing availability of large-scale data, genomics has become one of the largest research areas where ML is used [71]. The capacity of traditional statistics is often limited without the support of ML, especially in “multi-omics,” where multiple modes of genomics data, such as DNA-seq, RNA-seq, ChIP-seq, proteomics, and metagenomics, are analyzed together.

ML is used in genomics in multiple ways. For example, ML can be used to predict a certain gene’s expression level given the corresponding DNA-seq information. Genome-wide association studies (GWAS) that aim to identify genetic variants associated with a medical condition of interest, frequently involve ML algorithms such as neural networks and random forests [72]. Zou et al [73] provide more examples of deep learning applications to genomics.

The usefulness of ML as an auxiliary tool should not be underestimated. The overwhelming number of genes is often screened using the variable importance of random forests before downstream analyses. High-dimensional features can be reduced using autoencoders [74,75] to lower-resolution data, which can then be analyzed using traditional statistical analysis tools. Graphical illustrations of the data can also be created through 2D or 3D summaries using algorithms such as tSNE and PCoA [76,77].

Despite its broad capacity in genomics research and ability to handle high-dimensional data, ML has limitations. In genomics data, the number of features outnumbering the sample size, or high dimensionality, is a commonly seen attribute; even with the use of ML, the relatively small sample size can cause reliability and reproducibility issues.

### Precision Medicine

As previously mentioned, the goal of the research question of interest may be to indirectly support clinical decision-making. RL is a subset of ML that explicitly provides recommendations for decision-making at sequential time points. PM is a field where such algorithms can be applied to make treatment recommendations for individuals according to their unique characteristics.

PM starts from patient heterogeneity, where reactions to treatment vary from patient to patient [78]. For many illnesses, no panacea exists. PM seeks to recommend different treatment options for unique individuals based on their characteristics; this is formally called individualized treatment rules (ITRs) [79,80]. ITR forms the basis of PM by providing the best treatment recommendations tailored for each patient, as treatment effects can be heterogeneous among individuals. These rules are best identified with rich information about patients such as sociodemographic, clinical, and genomic data. Recently, a wealth of ITR methods have been developed [79-82].

Health care professionals and clinicians are often faced with treating a patient multiple times based on changes in response. For example, researchers can plan adaptive intervention programs for weight loss where later interventions are adjusted depending on responses to the previous treatment [83]. Such dynamic strategies are called dynamic treatment regimes (DTRs) [84,85]. DTRs aim to provide tailored decisions over more than 1 time point based on subject characteristics and their evolving contexts so that a long-term outcome of interest is optimized. The literature on this subtopic is fast-growing [86-88].

For example, patients with cancer may be given frontline chemotherapy followed by a salvage treatment if the response to the initial treatment is not successful [89]. A DTR can then be used to account for potential changes in a patient so that optimal recommendations can be made for each patient for unique stages in their disease to optimize a long-term outcome of interest, such as patient survival. An estimation of such DTR may require a large sample size. To address this issue, investigators can design a multistage randomized trial in an adaptive way through a sequential multiple assignment randomized trial (SMART) [83,90]. For instance, if a patient responded well to the first treatment, increasing the dose may not be particularly effective, and assigning a continued or decreased dose would be worth exploring. ITR and DTR are decision-support tools that provide the best treatment recommendations for patients.

SMARTs are an adaptive study design approach to finding a DTR. While SMARTs are used only for a fixed number of time points, the number of decision time points could be arbitrarily large for some problems. This is formally called an infinite-horizon setting. For example, artificial pancreas programs decide the amount of insulin infused every minute, so that numerous actions are taken even during a day [91]. A class of DTRs that provide essentially continuous recommendations is called just-in-time adaptive intervention [92]. A Markov decision process (MDP) is often used for these problems. MDP is a class of dynamic decision rules that base their decision only on the current state information, not necessarily depending on the history of the change. V-learning [93] is an example of such an infinite-horizon DTR that uses MDP structure.

PM has a strong connection with ML. Treatment effects are often dictated by thousands of patient characteristics, such as sociodemographic, genetic, clinical, and behavioral factors. Genetic factors alone are high-dimensional and can contain millions of traits. The goal of PM is to recommend the best treatment for a patient given their unique characteristics by providing them with an ITR. For example, a clinician may be interested in delineating an optimal ITR for each patient that best achieves cancer remission [94].

## Limitations and Optimizations

ML models trained on data that inaccurately represent the population cause fundamental issues such as biased prediction and suboptimality of decisions. Data, where the healthy population is poorly represented, may not be used to make a conclusion for the general population without adjustment. As ML can be used to support decision-making processes, it is also crucial that these decisions arising from the data are not discriminatory toward certain populations [95]. In the ML world, this term is called fairness. When the underlying data are biased, the ML algorithms that are trained on such data may produce biased results, which can lead to inaccurate predictions or withholding of resources. Bias in data may result from measurement bias, representation bias, and sampling bias, among others [96,97]. As an example, unbalanced gender data in the medical imaging field has led to algorithmic underperformance [98]. This discussion should be considered in the data quality assessment when planning data collection.

## Advanced Concepts

### Transformers

Transformers in the context of NLP are a type of deep learning model architecture that was first introduced by Vaswani in 2017 [63] and have outperformed other model types such as neural networks in both language generation and language interpretation [99]. Transformer models have a unique self-attention mechanism where the model can weigh the importance of different pieces of input data.

### Transfer Learning

Transfer learning is the process of taking knowledge from one task and applying it to a different task. This can be useful in several scenarios, such as when training data for models can be difficult to collect or it is computationally expensive to train a new model. In such cases, a pretrained model used in one task can then be trained using data from a different but related task, and the information learned from the previous task can be useful in the new task. This can have reduced data requirements and improved performance as opposed to training a brand-new model, especially when using large, pretrained, publicly available models [100].

### Reinforcement Learning

RL is a type of ML focused on training an algorithm to make sequential decisions in potentially changing environments to maximize a cumulative reward [101]. An agent, or decision maker, will receive some quantification of the current environment, also known as the state; the agent will then take an action that will change the state of the environment. The value associated with taking the action and transitioning to the next state is quantified by a “reward”; the agent should choose actions to maximize long-term reward. The goal may be to find the optimal sequence of actions to take to maximize the long-term reward. An application of this is decision support, which has previously been discussed. Other applications of RL range from PM to the development of self-driving cars to financial trading to the creation of ChatGPT. Those interested in the applications of RL in health care should see reference [102] for examples of use and [103] for guidelines of use.

## Outlook

The field of ML has a vast trove of tools and resources for use. Its potential, though impressive and exciting, can also be a drawback, as the inherent flexibility in the analysis process gives room for the researcher to arbitrarily or questionably choose methods that result in overfitting and false discovery. This paper has provided a framework for steps involved in using ML in research, discusses analyses for specialized data formats, reviews decision support and bias in ML, and introduces PM, a popular field in the health research domain. Consulting ML experts throughout the process will not only streamline the analysis but also play a large role in legitimizing justifications for choice selections.

Furthermore, the paper has highlighted the importance of preplanned documentation to ensure transparency and foster credibility within the scientific community. The incorporation of concrete examples within the health care domain, in addition to the provision of techniques involved in specialized data types, illustrates the vast applications of ML methodologies and their potential impact in the health science field.

Overall, the specifics of different data types and the wide variety of research goals make it difficult to make more specific recommendation guidelines. However, major ML considerations and how to approach them are discussed, with specific examples. While this paper provides a general recommended research framework and major considerations for the use of ML, it is not comprehensive, as the aim was to provide a general overview of potential methods and considerations.

While ML has strong performance potential in a variety of situations, its use needs to be carefully planned through the aforementioned steps and justified to obtain the best results, as ML cannot overcome poor study design or data quality despite all its virtues. By acknowledging these limitations, the research community can better strive for high-quality data and reproducible results to continue driving innovation in society.

## Appendix

Multimedia Appendix 1 Supplementary information on general sample size calculations, model training, and model tuning and validation.

## Abbreviations

AI: artificial intelligence
ATE: average treatment effects
AUC: area under the receiver operating characteristics curve
DTR: dynamic treatment regime
EHR: electronic health record
GWAS: genome-wide association studies
ITE: individual treatment effects
ITR: individualized treatment rule
k-NN: k-nearest neighbors
LDA: linear discriminant analysis
MDP: Markov decision process
ML: machine learning
NLP: natural language processing
PCA: principal component analysis
PM: precision medicine
RL: reinforcement learning
SMART: sequential multiple assignment randomized trial
SVM: support vector machine
TNR: true negative rate

## Data Availability

Data sharing is not applicable to this article as no data sets were generated or analyzed during this study.
