# Supplementary material for: Machine Learning and Health Science Research: Tutorial
Source: J Med Internet Res. 2024 Jan 30;26:e50890. doi: 10.2196/50890 (PMC10865203; doi:10.2196/50890)
Supplement: Multimedia Appendix 1 [file jmir_v26i1e50890_app1.docx]

Supplementary Materials for “Machine Learning and Health

Science Research”

July 15, 2023

# A Sample Size Calculation in Machine Learning

ML models typically require a greater sample size than traditional models. However, there is no deterministic formula which fits every model and scenario. Researchers may also be faced with a growing sample size requirement when more parameters need to be estimated from the data.

In linear regressions with certain mild assumptions, for example, a four times larger sample size would yield a twice ($=\surd4$) smaller standard error—in statistics we say that the estimator converges at a rate of *n*. On the other hand, in order for a nonparametric regression model with 10 covariates to achieve a twice smaller error, at least a 128 times larger sample is required under a twice differentiable density assumption. The best possible rate of convergence for a nonparametric estimator is
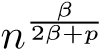
, where *p* is the dimension of the parameter, and 0 *<β<*∞ is some smoothness constant (Stone [16]).

## A.1 Mitigating the Large Sample Size Burden in Neural Networks

Many ML algorithms require a massive sample size. For example, Convolutional Neural Networks typically require 10,000-99,000 images per class in order to be trained to achieve a classification error rate of less than 20 % (Cho et al., 2015) [9]. However, there are techniques a researcher can implement in order to mitigate this burden. The first of which are data augmentation techniques. These techniques increase the amount of data by applying a transformation to the original data or images (Shorten and Khoshgoftaar, 2019 [13]). Moreover, one can augment data by adding noise to data sets during the training phase of the algorithm. This addition of noise leads to an increased sample size and reduction in classification error rate as seen in (Smilkov et al., 2017 [11]). This technique works particularly well in classification algorithms, specifically neural networks, and can greatly improve the evaluation metrics used (Tsinganos et al., 2020 [15]).

Transfer learning is particularly promising in that aspect. If one is able to find a suitable model that has already been trained, a relatively small sample size is needed thereafter (Xiao et al., 2019 [14]). Then, with the appropriate selection of a suitable model, one is able to reduce the sample size requirement significantly.

# B Tuning and Validation in Machine Learning

Assuming an appropriate model has been chosen, hyperparameter tuning and model validation becomes the next logical step for a ML practitioner. While this cannot be an exhaustive guide, we will cover hyperparameter tuning for tree models and for deep learning methods as these are the two most common models to use. This will be followed by a discussion of how to validate these models using a given sample, assuming collecting additional data is difficult.

The relatively high performance of these models is achieved by adequately tuning the hyperparameters. Thus, this tuning procedure is possibly the most critical element of ML pipelines. While it is often very challenging to tune models, overfitting is simultaneously an easy trap. Overfitting occurs when a model is overly flexible, fits the training data with high accuracy, but does not generalize well against previously unseen data (a high test-set error).

## B.1 Hyperparameter Tuning for Tree Models

Due to their relatively low computational cost as compared to most other ML methods, tree-based models are the most efficient to tune. The first conceptual point is to consider two broad parameters for tree-based learners - the complexity of each tree and the number of trees used. Number of trees should remain large. This is because fitting additional trees is computationally trivial, and as long as the individual trees are not too complex, taking the mean of a large number of properly parameterized models will not overfit the data. However, properly parameterizing the individual trees is not trivial (Qi, 2012 [18]).

Trees have three primary parameters - the number of features each tree considers, the number of leaves, and data required to create a leaf. These three parameters are the primary determinants of how flexible an individual tree is at fitting data. Number of leaves is the most intuitive. Each leaf is a terminal node in the tree and represents returning an output. Adding additional leaves will allow more complex functions to be fit. Each leaf defines a function of the data to return (usually a single value) across a range for each variable. So, more leaves mean more piecewise functions. This is the most important single parameter to be tuned.

The number of features each tree considers is an essential parameter to prevent overfitting. Since many models are being fit together (ensembled) in a tree model, restricting individual trees to only a subset of the features usually dramatically improves the model’s robustness. However, it is also important not to set this parameter too low so individual trees are not too weak to form a high-quality ensemble. Common ranges for this parameter are 0.5 (half the features available) to 1 (all the features available) and experimentation is encouraged.

The minimum number of observations required to create a split is another main check against overfitting. If this parameter is set too low, the model will be inclined to devote entire leaves to specific small sets of data that would otherwise give large amounts of loss, because there is little or no penalty to creating additional leaves to handle very specific subsets. This parameter is very similar to the maximum number of leaves however, and the two can frequently be tuned somewhat interchangeably.

Other tree-specific variables, such as resampling ratio, maximum depth, and number of trees, and parameters common to other statistical models can be tuned as well. In addition, each tree method (XGboost, lightGBM, Random Forests, etc.) has their own quirks. Learning the ins and outs of the specific package being used is critical. Finally, the speed at which Random Forests can be trained allows for some advanced methods in hyperparameter tuning. Python packages such as Optuna and Hyperopt can perform gradient descent over a hyperparameter grid in a specified range, using a validation set to analyze performance (Akiba, 2019 [12]; Bergstra and Cox, 2013 [20]).

## B.2 Hyperparameter Tuning for Deep Learning Models

This cannot serve as a full primer on deep learning but covers the basics. Assuming that a model has been selected, the next step is to choose an optimizer. Optimizers have two broad categories— those with and without momentum. Momentum formulations add some fraction of the direction of the last update step to the current update. Adam, a version of RMSprop with momentum, is the standard optimizer for deep learning applications and is very useful for navigating high-dimensional parameter landscapes (Kingma and Ba, 2014 [21]).

While Adam is good enough for most purposes, certain other optimizers are worth trying. Stochastic gradient descent and RMSprop are less likely to overfit on complex datasets due to the lack of momentum. Adadelta, Adamax, and NAdam are also worth trying due to the different formulation of the momentum mechanism and their mild tweaks to the gradient descent formula (Zeiler, 2012 [19]; Dozat, 2016 [10]). Optimizers are also paired with a learning rate. 0.001 is a standard starting value. A higher rate is less able to optimize the target function, while lower rates produce better optimizers at increased risk of overfitting. Optimizers are usually somewhat robust to the choice of learning rate; especially optimizers with momentum since they adjust the learning rate as they train. Learning rates can also be adjusted dynamically as the model trains. The two most common methods are a plateau decrease and a warmup/warm down routine.

Decreasing on plateau automatically drops the learning rate by a ratio, *R*, when loss on a validation set does not improve for *N* iterations. This helps to find an improved local optimum. Especially when the loss landscape is complex, local optimums can also be complex to find. This usually works well because a generally good parameter neighborhood has been found using a high learning rate, and then can be fine-tuned using the decreased learning rate. This of course comes at the risk of overfitting. Warm up/warm down works by setting a rapidly increasing learning rate early, followed by an exponentially decaying rate. This works to find a broadly good region across a large region of the parameter landscape, then finding a local minimum within that region using the exponentially decaying learning rate. Especially for complex datasets, these two techniques usually improve validation loss significantly over a flat learning rate.

Unless budget is high or time is unconstrained, it is usually not feasible to refit many times to find the best combination of parameters. Fitting for half or a quarter of the maximum number of epochs is usually enough to get a good picture of how a change to model learning behaviors.

## B.3 *K*-Fold Cross Validation

Splitting data into subsets is the best way to estimate model performance on out-of-sample data. *K*-fold cross validation is the most robust method to accomplish this (Varma and Simon, 2006 [17]). For *K*-fold cross-validation, we split a sample into *K* equal-sized subsamples, or “folds”. Of these folds, we treat one as the test set, one as an optional validation set, and the rest as the training set. While *K* is not fixed, 5 and 10 are the most common. This provides the best proxy for how a change in hyperparameters will perform on novel data and allows more efficient hyperparameter tuning at the cost of massive computation.

In stratified *K*-fold cross-validation, a more advanced, but also more accurate version of basic *K*-fold, the partitions are selected so that the distribution of response variables and covariates is similar across each fold. For example, in the case of binary classification, this means that each partition contains roughly the same proportions of the two types of class labels. This is especially important in ML when there may be a very complex target variable, and there may be drastically under sampled classes (Varma and Simon, 2006 [17]).

## B.4 Sample Splitting for High Dimensional Data

For high dimensional models, it is not possible to perform *K*-fold cross validation due to computational costs. We must instead return to a simple train/test split. We will ignore peculiarities such as time series and other specific classes of datasets, and instead focus on general principles. First, the stratified split or simple random split are the two most used and most robust methods to split a sample and should always be the default in absence of a special circumstance, such as a time series problem. However, with complex data, there can be significant issues in evaluating the effectiveness of a proposed sample split.

In large or complex datasets that require ML, there can be extremely complex correlation structures. This goes far beyond simple linear correlation, and as both complexity and size of data grows, the likelihood of contamination between inputs and targets, as well as mis-selection of train, test, and validation sets, grows dramatically.

We present a simple set of tricks to handle this issue. Assuming training testing and validation sets have been selected, training a smaller model to predict the set membership of observations can easily expose any issues. One possible trick is to label points according to the set they come from and evaluate the accuracy of the model. As long as accuracy at predicting the set is not very high, we can say that the splitting method was effective.

Researchers should also always be aware that as the number of features grows, the likelihood of target leakage grows. Target leakage is where the response variable is accidentally exposed in the training data and allows for outsized model accuracy. While this is a risk in any dataset, it is much more of a risk in the high-dimensional and often unstructured datasets common in ML. For this reason, it is important to be even more diligent about preventing any accidental leakage between the target variable and the features.

# C Model Training in Machine Learning

The goal of model training is to minimize the error between the predicted outcome and the actual outcome, which is a key concept used in the ML field and in traditional statistics. Recall, that there is not always a clear distinction between ML models and traditional statistical models. The loss function, sometimes referred to as the “cost function” is a mathematical way to quantify your model performance regarding the predicted outcome vs the actual outcome. To improve model performance, the loss function should be minimized, which indicates that your predicted values align with the true outcome. Therefore, model parameters should be tuned and adjusted on the training and validation (but not test) sets until the loss function is minimized through an optimization algorithm. An optimization algorithm, such as gradient descent, is used to find the minimum of the loss function, which adjusts model parameters in the direction of the gradient of the loss function step-by-step to minimize loss through an iterative process until a pre-specified convergence criteria is met.

In a simple linear regression, the loss can be thought of as the distance between the actual y-values, and the predicted y-values from the fitted model. A common loss function used in the regression setting is the mean squared error, which outputs the average squared distance between the predicted value and actual value. Other popular loss functions in the regression setting include L2 and L1 loss in penalized regression models, where the L2 loss is used in ridge regression, and the L1 loss is used in lasso regression. Additional examples of loss functions in regression settings include mean absolute error, Huber loss, and quantile loss among many others.

Loss functions can be employed in classification settings as well and tend to measure dissimilarity between the distributions of the predicted labels and true class labels. Common loss functions include cross entropy, which is also known as logarithmic loss, hinge loss, and Kullback-Leibler Divergence loss.

Models that have small loss function values on the training data, however, may also be overfit, and therefore may not generalize well when applied to testing data. This concept is known as the “bias-variance trade-off”. A model with high variance will be overfit, and captures underlying noise in the training data, which will not reflect well when applied to testing data. A model with high bias will be underfit and will have poor performance on training data as well as testing data.

Choosing a loss function will depend on the goal of your ML algorithm (regression vs classification), model regularization goals, prevalence of outliers, and other task-specific considerations.
